# Keratoscleritis and Choroidal Exudative Detachment in the Setting of Ocular Pyoderma Gangrenosum

**DOI:** 10.1155/crop/2166994

**Published:** 2026-03-11

**Authors:** Jeffrey Bodeen, Lulwa El Zein, Justin J. Yamanuha, Wassef Chanbour

**Affiliations:** ^1^ Department of Ophthalmology, University of Missouri School of Medicine, Columbia, Missouri, USA, missouri.edu; ^2^ Department of Ophthalmology and Visual Neurosciences, University of Minnesota, Minneapolis, Minnesota, USA, umn.edu

**Keywords:** choroidal effusion, keratitis and scleritis, ocular inflammation, postoperative corneal ulcer, pyoderma gangrenosum

## Abstract

**Purpose:**

The purpose of this study is to report a rare case of ocular pyoderma gangrenosum (PG) presenting as keratoscleritis with choroidal effusion following cataract surgery and to highlight its clinical recognition and management.

**Methods:**

A 79‐year‐old man developed a corneal ulcer 2 days after uncomplicated phacoemulsification with intraocular lens implantation. Initial management with topical vancomycin and ceftazidime failed. Comprehensive systemic workup including TPMT, HLA‐B51, ANCA, ANA, rheumatoid factor, viral serologies, and syphilis testing was performed. Cultures for bacteria, fungi, and *Acanthamoeba* were negative. The history of PG confirmed by prior skin biopsy was elicited. Corticosteroid therapy was initiated.

**Results:**

The patient′s corneal ulcer, keratoscleritis, and choroidal effusions resolved within 2 weeks of treatment with high‐dose topical and oral corticosteroids. Visual acuity improved from hand motion to 20/30, and intraocular pressure normalized. No infectious etiology was identified, and rapid response to steroids excluded active herpetic keratitis.

**Conclusion:**

Ocular PG can manifest as postoperative keratoscleritis with choroidal effusions. Negative cultures and poor response to antimicrobials should prompt consideration of immune‐mediated keratitis. Early recognition and timely corticosteroid therapy can preserve vision. Preoperative risk assessment and perioperative steroid prophylaxis may prevent postoperative inflammatory complications in patients with known PG.

## 1. Introduction

Pyoderma gangrenosum (PG) is an autoimmune neutrophilic dermatosis that typically presents as a pustule, vesicle, or nodule that rapidly expands and ulcerates [1]. Several clinical variants exist: the ulcerative (classic) form, the pustular form, characterized by painful sterile pustules with an erythematous halo, and the bullous form, featuring rapidly evolving vesicles and bullae with central necrosis [2]. A vegetative form may also occur, presenting with slowly progressive superficial ulceration and undermined borders. PG can affect peristomal, genital, or extracutaneous sites—including the lungs, heart, central nervous system, and other organs—and may occasionally present without cutaneous involvement [2].

Although rare, atypical presentations of PG can involve ocular structures. Ocular PG most frequently occurs in patients with underlying systemic diseases such as multiple sclerosis, inflammatory bowel disease, rheumatoid arthritis, or myeloproliferative disorders [3–6]. Ophthalmic involvement most commonly affects the eyelids and adnexa, presenting as painful nodules or ulcerations [4]. In rare cases, the ocular surface and deeper structures—including the sclera and cornea—may be affected, often in association with systemic disease [3–6].

Here, we present a rare case of ocular PG manifesting as keratoscleritis and choroidal effusion triggered by cataract surgery, highlighting the importance of recognizing immune‐mediated etiologies in postoperative keratitis. Written informed consent to participate and publish information and images was obtained from the participant.

## 2. Case

**Figure 1:**
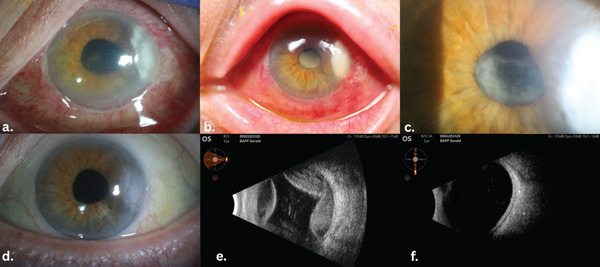
(a) Temporal corneal ulcer located at the cataract surgery incision site with a 4+ anterior chamber reaction, 5 days after cataract surgery. (b) Enlarged and denser ulceration with formation of a fibrinous pupillary membrane, 2 days after admission. (c) Dense, inactive pupillary membrane 2 weeks after initiation of corticosteroid therapy. (d) Healed ulceration with a residual temporal corneal scar. (e) Dense exudative choroidal effusion noted 2 days after admission. (f) Resolution of the choroidal effusion, 2 weeks following steroid treatment.

A 79‐year‐old man was referred to the emergency department of a tertiary care center 5 days after undergoing uncomplicated phacoemulsification cataract surgery with intraocular lens implantation in the left eye. Two days postoperatively, he was diagnosed with a corneal ulcer at the surgical wound, which progressively worsened despite treatment with topical vancomycin and ceftazidime. His medical history was significant for Type 2 diabetes mellitus complicated by diabetic polyneuropathy, Stage 3 chronic kidney disease, hyperlipidemia, anemia likely secondary to CKD, obesity–hypoventilation syndrome, fatty liver disease, congestive heart failure, and COPD. On presentation, best‐corrected visual acuity in the left eye was hand motion, and intraocular pressure was 32 mmHg. No afferent pupillary defect was noted. Slit lamp examination revealed 2+ conjunctival injection and chemosis, a 4 × 2 mm corneal infiltrate with an overlying epithelial defect, and 4+ cellular reaction in the anterior chamber (Figure 1a). A B‐scan demonstrated mild vitreous debris without retinal detachment. Gram stain showed 4+ white blood cells, and corneal cultures were obtained. Hourly fortified vancomycin (25 mg/mL) and tobramycin (14 mg/mL) eye drops were resumed. The patient was admitted to the intensive care unit for concurrent hypotension and hypoxic respiratory failure. Two days after admission, the corneal ulcer was noted to be worse with enlarged size and density of the stromal infiltrate, and fibrinous reaction was noted in the anterior chamber (Figure 1b). A repeat B‐scan demonstrated small to medium choroidal effusions with dense fluid but no retinal traction, detachment, or apposition (Figure 1e). A vitreous tap was deferred, as the clinical presentation was not consistent with typical endophthalmitis, which is usually characterized by pain and dense vitreous opacities, and due to the potential risk of wound leakage. By Day 5 of hospitalization, there was no improvement in the ulcer or choroidal effusions, and fibrin persisted in the anterior chamber. Bacterial, fungal, and *Acanthamoeba* cultures remained negative. In light of the lack of response, topical amphotericin and voriconazole were added hourly. A comprehensive systemic workup was initiated, including HLA‐B51, ANCA, QuantiFERON, rheumatoid factor, antinuclear antibody, *Treponema pallidum*, hepatitis B surface antigen, hepatitis C antibody, and HIV testing—all of which were negative. HSV‐1 and HSV‐2 IgG antibodies were positive. At this point, the patient′s history of PG—confirmed by skin biopsy 1 year earlier showing neutrophilic infiltrate and granulomatous inflammation—was noted by a family member. Two weeks after admission, antimicrobial therapy was discontinued, and the patient was started on prednisolone acetate 1% eye drops hourly and oral prednisone 60 mg daily. Two weeks after initiating corticosteroid therapy, both the corneal ulcer and choroidal effusions had resolved (Figure 1f). A pupillary membrane was noted (Figure 1c), and YAG laser membranectomy was performed (Figure 1d). One month after treatment, the patient′s visual acuity improved to 20/30, with an intraocular pressure of 15 mmHg. Steroid therapy was tapered over a 2‐month period, and there was no recurrence of ulceration 6 months after treatment was discontinued.

## 3. Discussion

Sterile keratitis should be suspected in cases of corneal ulceration unresponsive to broad‐spectrum antimicrobials and negative cultures [7]. In such settings, early recognition of immune‐mediated causes is essential to avoid unnecessary antimicrobial exposure, delay in definitive treatment, and potential vision loss. Although HSV serology was positive, the rapid and significant improvement after initiating corticosteroid therapy makes active herpetic keratitis unlikely, as such cases typically worsen with steroids alone. This case highlights an uncommon but clinically important presentation of ocular PG, manifesting as keratoscleritis with choroidal effusions following cataract surgery.

A 2017 systematic review classified ocular PG into four categories: (1) cutaneous periocular PG, (2) periocular PG with orbital involvement, (3) extracutaneous orbital/scleral PG, and (4) ocular disease associated with PG [7]. Our patient fits Category 3, with scleral and corneal involvement without cutaneous activity at presentation. Ocular PG likely reflects the same underlying dysregulated neutrophilic inflammatory response seen in cutaneous PG, often triggered by trauma (pathergy phenomenon) [7, 8]. This patient′s cataract surgery may have acted as a local trigger, leading to immune‐mediated keratitis and scleritis. Posterior extension of scleritis can result in choroidal involvement, including choroidal effusions, through inflammatory thickening of the choroid and increased vascular permeability [4, 5]. Choroidal effusions may be complicated with secondary hypotony, anterior chamber shallowing, angle closure, and exudative retinal detachment, resulting in permanent vision loss if it was not recognized and treated [9]. In the postoperative setting, choroidal effusions may also mimic more common entities such as endophthalmitis or suprachoroidal hemorrhage, potentially delaying appropriate therapy [9]. While choroidal effusions are well described in posterior scleritis, dense exudative choroidal effusion associated with PG has not been previously documented, making this case novel.

Multiple reports describe PG‐associated scleritis and keratitis [9–11]. Documented cases include scleritis occurring in patients with underlying systemic inflammatory diseases as well as nodular scleritis with concurrent corneal ulceration in the setting of ulcerative colitis. Similar to our patient, these cases demonstrated poor response to antimicrobial therapy but improved significantly with systemic corticosteroids.

Ocular PG is a diagnosis of exclusion [7]. Clinical suspicion should be high in patients with a history of PG presenting with postoperative keratitis unresponsive to antimicrobials, especially when cultures are negative. Due to the lack of specific histological markers [7] and the high risk of corneal wound leak in our case, a corneal biopsy was not performed. Early recognition can prevent unnecessary prolonged use of antibiotics and facilitate prompt initiation of immunosuppressive therapy. In our patient, initiation of high‐dose topical and systemic corticosteroids led to rapid resolution of both the corneal ulcer and choroidal effusions, and significant visual recovery.

Preoperative risk stratification and perioperative steroid prophylaxis should be considered in patients with a known history of PG to minimize postoperative inflammatory complications.

## 4. Conclusion

This case expands the spectrum of ocular PG and underscores the importance of including immune‐mediated etiologies in the differential diagnosis of postoperative keratitis, particularly when cultures are negative and clinical response is poor. It also highlights the potential for vision preservation when inflammation is promptly identified and treated with immunosuppression.

## Author Contributions

All listed authors made significant contributions; Jeffrey Bodeen contributed to data collection and draft writing. Lulwa El Zein, Justin J. Yamanuha, and Wassef Chanbour contributed to conceptualization, validation, supervision, and review.

## Funding

The authors did not receive direct funding for this project, but it was made during employment at the University of Minnesota.

## Conflicts of Interest

The authors declare no conflicts of interest.

## Data Availability

Data sharing is not applicable to this article as no datasets were generated or analyzed during the current study.
